# Impact of adverse childhood experiences, post-traumatic stress disorder, dissociative disorders, and depression on dementia risk: a prospective analysis of associations and mediation in the UK Biobank cohort

**DOI:** 10.1192/j.eurpsy.2025.10128

**Published:** 2025-10-29

**Authors:** Mia Maria Günak, Thomas Ehring, Vasiliki Orgeta, Frederick K. Ho

**Affiliations:** 1 Department of Psychology, Division of Clinical Psychology and Psychological Treatment, LMU Munich, Munich, Germany; 2 School of Health & Wellbeing, University of Glasgow, Glasgow, UK; 3 Division of Psychiatry, Faculty of Brain Sciences, UCL, London, UK; 4 German Center for Mental Health (DZPG), Munich, Germany

**Keywords:** Adverse childhood experiences (ACEs), post-traumatic stress disorder (PTSD), dissociative disorders, depression, dementia risk

## Abstract

**Background:**

Little is known about the interrelationships among adverse childhood experiences (ACEs), post-traumatic stress disorder (PTSD), dissociative disorders, depression, and dementia risk. We sought to investigate associations of ACEs, PTSD, dissociative disorders, and depression with incident dementia and explore whether these associations may be interrelated through mediation.

**Methods:**

This prospective cohort study used population-based UK Biobank data, including 502 355 participants recruited at 22 assessment centres who completed questionnaires, an interview, and physical assessments at baseline (2006–2010). Data are linked to participants’ electronic health records from primary care, hospital admissions, and death registers through November 30, 2022, and to the results of the UK Biobank online mental health survey (2016–2017). Cox regression and g-formula-based mediation analyses were used to examine associations between self-reported ACEs, self-reported PTSD symptoms, diagnosed PTSD, dissociative disorders, depression, and dementia.

**Results:**

In the final sample (*n* = 434 215, mean (SD) age 56.58 (8.07) years), ACEs (hazard ratio (HR)_1point_: 1.10; 95% CI 1.02–1.20), diagnosed PTSD (HR: 2.09; 95% CI 1.38–3.18), dissociative disorders (HR: 3.96; 95% CI 2.55–6.15), depression (HR: 2.17; 95% CI 2.05–2.30), and self-reported PTSD symptoms (HR_1point_: 1.09; 95% CI 1.06–1.11) were associated with increased dementia risk, after adjusting for sociodemographic characteristics. Self-reported PTSD symptoms explained 75.26% (*P* < .001) of the excess dementia risk associated with ACEs. Depression explained 4.51% (*P* = .02) of the ACEs-dementia link, 8.42% (*P* < .001) of the diagnosed PTSD-dementia link, and 10.29% (*P* < .001) of the dissociative disorders-dementia link.

**Conclusions:**

Individuals with ACEs, PTSD, dissociative disorders, or depression appear to be at increased risk of dementia, potentially through both shared and unique associations. However, these findings should be interpreted with caution due to potential limitations in statistical power.

## Introduction

Dementia is expected to become more prevalent as the global population ages [1]. Identifying modifiable risk factors to prevent or delay its onset and progression has been a major focus of dementia research [2]. Studies indicate that adverse childhood experiences (ACEs), including neglect and abuse [3, 4], are associated with an increased risk of all-cause dementia [5, 6]. Among the negative mental health consequences frequently linked to ACEs are depression [7], post-traumatic stress disorder (PTSD) [8], and dissociative disorders [9]. Although depression [2] and PTSD [10] have both been suggested as risk factors for dementia, no study to date has examined the role of dissociative disorders, which are frequently overlooked in research [11], nor the interrelationships among ACEs, PTSD, dissociative disorders, and depression in increasing dementia risk. Prior studies investigating the relationship between PTSD and incident dementia [12–19] have typically adjusted for depression, which is often comorbid with PTSD [20] and dissociative disorders [9]. The associations adjusted for depression, while attenuated, have remained significant. However, potential mediational relationships have not been systematically examined despite evidence of the frequent comorbidity and sequential occurrence of ACEs, PTSD, dissociative disorders, and depression.

The aim of our study was to use prospective data from a large cohort of the general population in the United Kingdom (UK) to investigate the associations between ACEs and PTSD, dissociative disorders, depression, and subsequent dementia. Additionally, we sought to explore the interrelationships among these exposures and their link to dementia via mediational analyses. Three research questions were investigated: 1) Are ACEs, PTSD, dissociative disorders, and depression each associated with incident all-cause dementia? 2) Do PTSD and dissociative disorders each mediate the association between ACEs and dementia? and 3) Is depression a mediator of the associations between ACEs and dementia, PTSD and dementia, and dissociative disorders and dementia?

## Methods

### Data and participants

We analysed data from the UK Biobank, which is a population-based prospective cohort study that included more than half a million participants. Between 2006 and 2010 (baseline), individuals aged 37–73 years attended one of 22 assessment centres across England, Scotland, and Wales to complete a self-administered touchscreen questionnaire and a face-to-face interview inquiring about various aspects of life, such as sociodemographics and lifestyle. Trained staff conducted physical assessments and collected biological samples. These baseline data are linked to electronic health records from primary care, hospital admissions, and death registers, with retrospective data coverage extending to at least 10 years before the UK Biobank baseline. At the time of our analysis in May 2024, data were available until November 30, 2022. In 2016 and 2017, approximately one-third (*n* = 157 329, 31.32%) [21] of the overall sample completed an online mental health questionnaire capturing symptoms of possible mental disorders, as well as items on ACEs, including neglect and abuse. The UK Biobank received ethics approval from the North-West Multi-centre Research Ethics Committee (21/NW/0157), and all participants provided written informed consent at baseline and were free to withdraw at any time. Further information about the UK Biobank protocol can be found online (https://www.ukbiobank.ac.uk).

We calculated age at baseline using the date of birth and date of assessment. Sex, ethnicity, highest attained level of education, sleep duration, weekly alcohol consumption, smoking status, cardiovascular diseases, and traumatic brain injury (TBI) were self-reported at baseline. The Townsend deprivation index was derived from area-based aggregated data on unemployment, car and home ownership, and household overcrowding [22]. Weekly physical activity was assessed using the validated International Physical Activity Questionnaire (IPAQ) [23]. We defined hypertension as a measured systolic blood pressure of at least 140 mmHg or self-reported prescription of antihypertensive medication at baseline. We specified the increasing risk of harm from alcohol consumption as 15–34 units per week for women and 15–49 units per week for men in accordance with the National Institute for Health and Care Excellence guidance [24]. Consumption below and above these ranges was considered lower and higher risk.

### Measures

#### Adverse childhood experiences

Information on ACEs was collected by the UK Biobank in its online mental health questionnaire using the validated Childhood Trauma Screener (CTS) [25], a shortened version of the Childhood Trauma Questionnaire (CTQ) [26]. Respondents rate five types of child maltreatment (i.e., sexual, emotional, and physical abuse; emotional and physical neglect) on a five-point Likert scale. Cut-off scores were used to determine the presence or absence of each type of ACE, resulting in a total number of ACE types experienced (0–5) [25]. In our analyses, we took into account the time points at which ACEs were measured.

#### PTSD, dissociative disorders, and depression

We identified diagnoses of PTSD, dissociative disorders, and depression through linked electronic health records. The date of diagnosis was based on the first recorded occurrence in primary care, hospital admissions, or death registers. We included only those participants who received any of the mentioned diagnoses before a dementia diagnosis or censoring (i.e., last date of observation). The comparison group comprised participants without any exposure diagnoses before any dementia diagnosis or censoring. We used *International Classification of Diseases, Tenth Revision* (ICD-10) [27] codes to identify a diagnosis of PTSD (F43.1), dissociative disorders (F44.x, F48.1), or depression (F32.0 to F32.3, F32.8, F32.9, F33.0 to F33.3, F33.8, F33.9).

An adapted five-item version of the PTSD Checklist – Civilian Version (PCL-C) [28] included in the online mental health survey was used as a self-report measure of past-month PTSD symptoms. Items assessed intrusive thoughts, distress when reminded of a trauma, avoidance, feeling distant from others, and irritability [21]. These were rated on a five-point Likert scale and summed to a total severity score. Participants who completed the adapted PCL-C were included if any dementia diagnosis was recorded only after the online mental health survey or not at all, until censoring.

#### Dementia

We also ascertained all-cause dementia incidence and date of first diagnosis through the linked electronic health records using the following ICD-10 [27] codes: A81.0, F00.x, F01.x, F02.x, F03, F05.1, F10.6, G30.x, G31.0, G31.1, G31.8.

### Statistical analyses

We used Cox proportional hazard models to estimate the associations of ACEs, PTSD, dissociative disorders, and depression with incident all-cause dementia, reporting hazard ratios (HR) and 95% confidence intervals (CI). The outcome variable consisted of the event status and time-to-event. We adjusted our main model for age, sex, ethnicity (White versus Asian, Black, Mixed, or Other), education level (with vs. without a college or university degree), and Townsend deprivation index (≥ vs. < median) as potential confounders. These factors have been shown to influence the risk of dementia, trauma-related conditions, and depression [2, 29, 30]. We tested proportional hazard assumptions using statistical tests based on Schoenfeld residuals.

We conducted two sensitivity analyses. First, we repeated the main analyses but with an additional adjustment for lifestyle factors and medical comorbidities (i.e., sleep duration, weekly alcohol consumption, smoking status, weekly physical activity, cardiovascular conditions, TBI, and hypertension). These variables could be mediators between exposures and dementia and were therefore only adjusted for in sensitivity analyses. Second, we repeated the first sensitivity analysis but with an additional adjustment for depression in the models in which ACEs, PTSD, or dissociative disorders were the exposures. In the model in which depression was the exposure, we repeated the first sensitivity analysis but with an additional adjustment for ACEs, PTSD, and dissociative disorders combined (due to small numbers in the PTSD and dissociative disorder groups). Given the relatively low prevalence of diagnosed PTSD, we performed a post-hoc analysis of the association between self-reported PTSD symptoms and subsequent dementia incidence. Hereinafter, “PTSD” refers to diagnoses from electronic health records, while “self-reported PTSD symptoms” refer to self-reported PTSD symptoms from the online mental health survey. Additionally, we conducted two post-hoc subgroup analyses: one comprising participants with depression but no PTSD or dissociative disorders, and another comprising participants with depression but no ACEs, PTSD, or dissociative disorders.

We implemented two mediation models [31]. First, we examined whether PTSD (diagnosis [binary] and symptoms [continuous]) and dissociative disorders (binary) mediated the relationship between ACEs (ordinal) as the exposure and incident all-cause dementia (binary) as the outcome. Second, we examined whether depression (binary) mediated the relationship between trauma-related conditions (ACEs, PTSD, self-reported PTSD symptoms, or dissociative disorders) as the exposure and incident all-cause dementia as the outcome. We regressed dementia (outcome) on the potential mediator, primary exposure variable, and covariates (i.e., age, sex, ethnicity, education level, area-based deprivation) using logistic models for binary mediators and multiple linear models for continuous mediators. We also regressed the potential mediators on the primary exposure and covariates. We then combined the results of the outcome and mediator regression models using the g-formula with bootstrapping to estimate the proportion of the total effect mediated. We considered the time sequence, coding the primary exposure and mediators as ‘present’ only if they occurred before the mediator and dementia diagnosis, respectively.

We included participants in the analyses only if they had complete data on all variables, except for the online survey measures (due to power issues). All analyses were conducted between January 2023 and May 2024 using R (version 4.2.0; details in Supplementary Material).

## Results

### Sample characteristics

**Figure 1.**
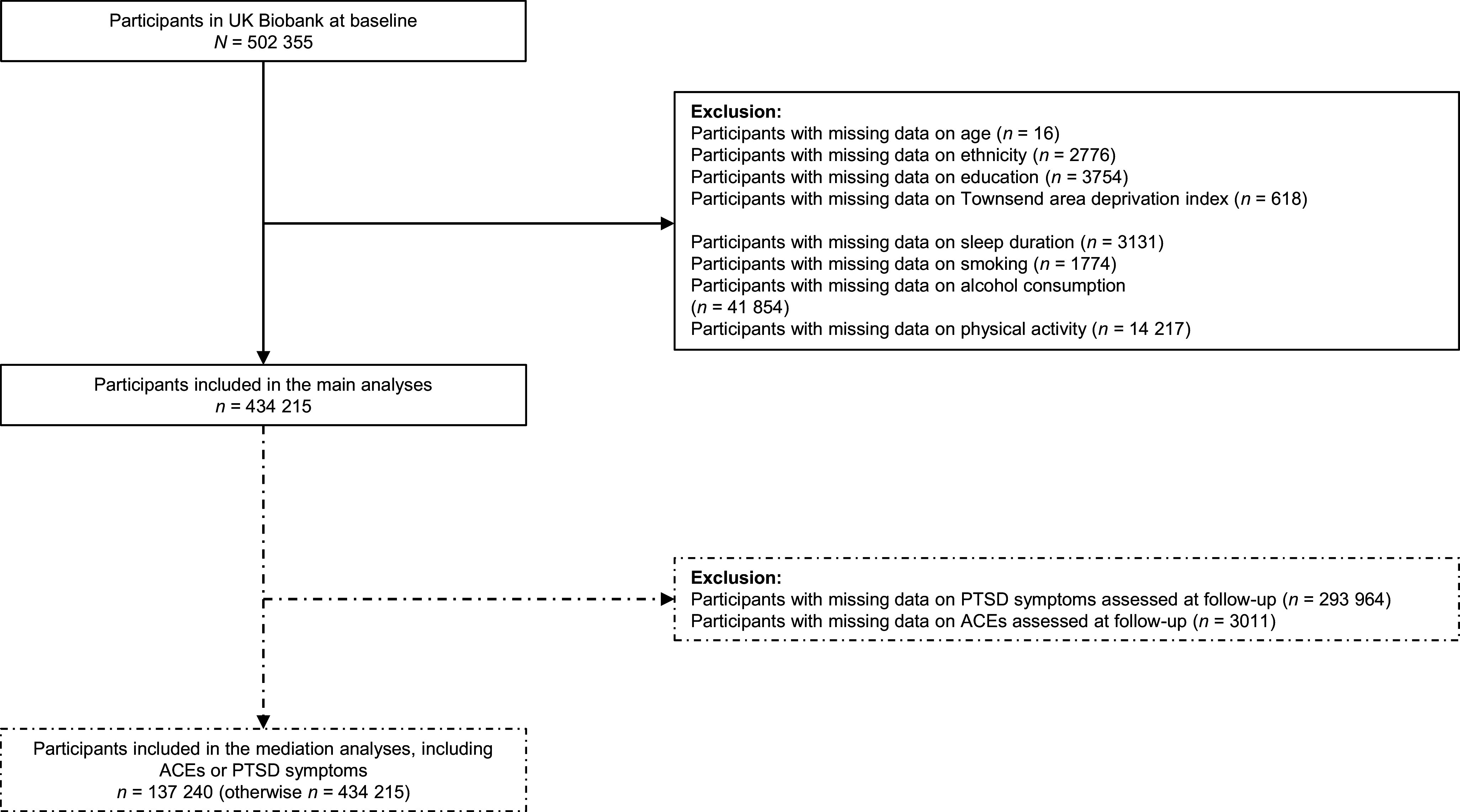
Diagram of participants included in the analyses. ACEs, adverse childhood experiences; *N*, sample size; PTSD, post-traumatic stress disorder.

After excluding participants with missing values for any of the covariates included in the analyses (*n* = 68 140; Figure 1), the final cohort comprised 434 215 participants (mean (SD) age 56.58 (8.07) years, 53.52% female; Table 1). Median follow-up was 13.66 years (IQR = 12.87–14.39). In total, 941 (0.22%) participants were diagnosed with PTSD, 325 (0.07%) with any dissociative disorder, and 44 140 (10.17%) with depression before any dementia diagnosis or censoring. People with any of these diagnoses were generally less educated, more deprived, more often current smokers, and less physically active, and reported more sleep duration deviations and cardiovascular diseases (Table 1). Approximately one-third (*n* = 45 536) of the participants who took part in the online mental health survey (*n* = 140 251) experienced at least one type of ACE. Of these ACEs, emotional neglect was the most common (66.26%), followed by emotional abuse (27.82%), sexual abuse (26.37%), physical abuse (23.97%), and physical neglect (16.66%; Supplementary Table 1). Dementia developed in 266 individuals with ACEs (0.58%), 22 of those with PTSD (2.34%), 20 of those with any dissociative disorder (6.15%), and 1397 of those with depression (3.16%). In comparison, dementia occurred in 6703 individuals without PTSD, dissociative disorders, or depression (1.72%), and in 454 individuals without these diagnoses or ACEs (0.52%; Supplementary Tables 2 and 3).

**Table 1.** Baseline characteristics by group

	Overall (*n* = 434 215)	Comparison Group^a^ (*n* = 389 516)	Comparison Group 2^b^ (*n* = 86 496)	ACEs^c^ (*n* = 45 536)	PTSD (*n* = 941)	Dissociative Disorders (*n* = 325)	Depression (*n* = 44 140)	Depression Only^d^ (*n* = 43 452)	Depression Only 2^e^ (*n* = 5489)
Age, mean (SD)	56.58 (8.07)	56.65 (8.07)	56.25 (7.69)	55.52 (7.75)	52.81 (8.00)	55.26 (8.01)	55.98 (8.04)	56.03 (8.04)	55.78 (7.60)
Age groups, *n* (%)									
37–50	112 822 (25.98)	100 206 (25.73)	21 980 (25.41)	12 983 (28.51)	407 (43.25)	92 (28.31)	12 419 (28.14)	12 126 (27.91)	1449 (26.40)
51–60	153 432 (35.34)	137 068 (35.19)	34 009 (39.32)	18 336 (40.27)	332 (35.28)	133 (40.92)	16 163 (36.62)	15 908 (36.61)	2274 (41.43)
61–73	167 961 (38.68)	152 242 (39.08)	30 507 (35.27)	14 217 (31.22)	202 (21.47)	100 (30.77)	15 558 (35.25)	15 418 (35.48)	1766 (32.17)
Sex, *n* (%)									
Female	232 395 (53.52)	203 870 (52.34)	46 154 (53.36)	26 431 (58.04)	439 (46.65)	230 (70.77)	28 257 (64.02)	27 866 (64.13)	3626 (66.06)
Male	201 820 (46.48)	185 646 (47.66)	40 342 (46.64)	19 105 (41.96)	502 (53.35)	95 (29.23)	15 883 (35.98)	15 586 (35.87)	1863 (33.94)
Asian, Black, Mixed, or Other ethnic background, *n* (%)^f^	23 078 (5.31)	21 104 (5.42)	1768 (2.04)	2028 (4.45)	97 (10.31)	15 (4.62)	1934 (4.38)	1863 (4.29)	78 (1.42)
Education, *n* (%) College or university degree	146 714 (33.79)	134 553 (34.54)	41 683 (48.19)	20 206 (44.37)	261 (27.74)	78 (24.00)	12 014 (27.22)	11 823 (27.21)	2254 (41.06)
Townsend deprivation index >=Mdn^g^	213 920 (49.27)	188 502 (48.39)	36 751 (42.49)	22 736 (49.93)	599 (63.66)	203 (62.46)	25 083 (56.83)	24 628 (56.68)	2537 (46.22)
Sleep duration, *n* (%)									
< 6 hours	23 186 (5.34)	18 912 (4.86)	2444 (2.83)	2289 (5.03)	165 (17.53)	51 (15.69)	4203 (9.52)	4063 (9.35)	299 (5.45)
6–9 hours	403 188 (92.85)	364 723 (93.63)	83 376 (96.39)	42 619 (93.59)	729 (77.47)	246 (75.69)	38 001 (86.09)	37 503 (86.31)	5058 (92.15)
> 9 hours	7841 (1.81)	5881 (1.51)	676 (0.78)	628 (1.38)	47 (4.99)	28 (8.62)	1936 (4.39)	1886 (4.34)	132 (2.40)
Smoking, *n* (%)									
Current	44 596 (10.27)	37 035 (9.51)	5108 (5.91)	4176 (9.17)	206 (21.89)	48 (14.77)	7473 (16.93)	7311 (16.83)	536 (9.76)
Never	236 450 (54.45)	215 457 (55.31)	52 036 (60.16)	23 502 (51.61)	430 (45.70)	179 (55.08)	20 717 (46.93)	20 392 (46.93)	2967 (54.05)
Former	153 169 (35.27)	137 024 (35.18)	29 352 (33.93)	17 858 (39.22)	305 (32.41)	98 (30.15)	15 950 (36.14)	15 749 (36.24)	1986 (36.18)
Alcohol consumption per week, Mdn (IQR)	10.50 (2.76–22.50)	11.25 (3.00–22.65)	12.00 (4.50–22.50)	10.65 (3.00–22.20)	7.20 (1.50–21.00)	1.50 (1.38–11.40)	8.70 (1.50–20.40)	8.70 (1.50–20.40)	9.15 (2.35–20.40)
Risk of harm from alcohol, *n* (%)									
Higher risk	30 881 (7.11)	27 345 (7.02)	4872 (5.63)	3089 (6.78)	93 (9.88)	12 (3.69)	3490 (7.91)	3433 (7.90)	328 (5.98)
Increasing risk	143 439 (33.03)	131 500 (33.76)	31 507 (36.34)	15 100 (33.16)	242 (25.72)	53 (16.31)	11 782 (26.69)	11 646 (26.80)	1692 (30.83)
Lower risk	259 895 (59.85)	230 671 (59.22)	50 117 (57.94)	27 347 (60.06)	606 (64.40)	260 (80.00)	28 868 (65.40)	28 373 (65.30)	3469 (63.20)
Physical activity, minutes per week, Mdn (IQR)	1582.00 (678.00–3332.00)	1605.00 (693.00–3342.00)	1572.00 (720.00–3066.00)	1590.00 (699.00–3172.00)	1413.00 (438.00–3333.00)	1050 (311.00–3066.00)	1386.00 (495.00–3150.00)	1386.00 (495.00–3150.00)	1386.00 (594.00–2892.00)
Cardiovascular diseases, *n* (%)	28 609 (6.59)	24 304 (6.24)	3547 (4.10)	2229 (4.90)	104 (11.05)	57 (17.54)	4235 (9.59)	4146 (9.54)	284 (5.17)
Traumatic brain injury, *n* (%)	1399 (0.31)	1138 (0.29)	208 (0.24)	130 (0.29)	8 (0.85)	4 (1.23)	198 (0.45)	189 (0.43)	15 (0.27)
Hypertension, *n* (%)	217 304 (50.05)	195 526 (50.20)	39 585 (45.77)	19 649 (43.15)	453 (48.14)	165 (50.77)	21 503 (48.72)	21 166 (48.71)	2406 (43.83)
Depression, *n* (%)	44 140 (10.17)	NA	NA	5381 (11.82)	526 (55.90)	177 (54.46)	44 140 (100.00)	43 452 (100.00)	5489 (100.00)
PTSD, *n* (%)	941 (0.22)	NA	NA	111 (0.24)	941 (100)	19 (5.85)	526 (1.19)	NA	NA
Dissociative disorders, *n* (%)	325 (0.07)	NA	NA	47 (0.10)	19 (2.02)	325 (100.00)	177 (0.40)	NA	NA
Self-reported PTSD symptoms, mean (SD)	7.96 (3.06)	7.75 (2.78)	7.31 (2.25)	8.99 (3.80)	15.05 (7.09)	11.66 (5.67)	10.27 (4.67)	10.20 (4.59)	8.92 (3.62)

Abbreviations: ACEs, adverse childhood experiences; IQR, interquartile range; Mdn, median; *N*, sample size; NA, not applicable; PTSD, post-traumatic stress disorder; SD, standard deviation; TBI, traumatic brain injury.

a “Comparison group” refers to participants without PTSD, dissociative disorders, or depression (PTSD–/Dissociative disorders–/Depression–).

b “Comparison group 2” refers to participants without PTSD, dissociative disorders, or depression, who self-reported as part of the online mental health survey that they had no ACEs (ACEs–/PTSD–/Dissociative disorders–/Depression–).

c “ACEs” group refers to participants who self-reported that they had at least one type of ACEs, as part of the online mental health survey (ACEs+).

d “Depression only” group refers to participants with depression but without PTSD or dissociative disorders (Depression+/ PTSD–/Dissociative disorders–).

e “Depression only 2” group refers to participants without PTSD, dissociative disorders, or depression, who self-reported as part of the online mental health survey that they had no ACEs (Depression+/ACEs–/PTSD–/Dissociative disorders–).

f Including Asian or Asian British, Black or Black British, Chinese, Mixed, or Other ethnic group.

g Median = −2.135.

### ACEs, PTSD, dissociative disorders, depression, and dementia

After adjusting for sociodemographic characteristics, we found that each additional type of ACE experienced was associated with a 10% increase in the risk of developing dementia (HR_1point_: 1.10; 95% CI 1.02–1.20; *P* = .018). The risk of all-cause dementia was 2.09 to 3.96 times higher in people with diagnosed PTSD (HR: 2.09; 95% CI 1.38–3.18; *P* < .001), any dissociative disorders (HR: 3.96; 95% CI 2.55–6.15; *P* < .001), or depression (HR: 2.17; 95% CI 2.05–2.30; *P* < .001) compared with people without the respective diagnosis (Table 2).

**Table 2.** Unadjusted and adjusted risk of dementia by group

	HR (95% CI)			
Model	Unadjusted	Main model^a^	Sensitivity analysis 1^b^	Sensitivity analysis 2^c^
ACEs (*n* = 45 536)	1.03 (0.95, 1.11), *P* = .50	1.10 (1.02, 1.20), *P* = .018*	1.09 (1.00, 1.19), *P* = .039*	1.07 (0.99, 1.17), *P* = .10
PTSD (*n* = 941)	1.27 (0.84, 1.93), *P* = .30	2.09 (1.38, 3.18), *P* < .001**	0.94 (0.45, 1.97), *P* = .90	0.68 (0.32, 1.44), *P* = .30
Self-reported PTSD Symptoms (*n* = 140 251)	1.03 (1.01, 1.05), *P* = .005*	1.09 (1.06, 1.11), *P* < .001**	1.08 (1.06, 1.11), *P* < .001**	1.07 (1.05, 1.10), *P* < .001**
Dissociative Disorders (*n* = 325)	3.46 (2.23, 5.36), *P* < .001**	3.96 (2.55, 6.15), *P* < .001**	1.73 (0.84, 3.58), *P* = .14	1.37 (0.65, 2.86), *P* = .40
Depression (*n* = 44 140)	1.91 (1.80, 2.02), *P* < .001**	2.17 (2.05, 2.30), *P* < .001**	2.00 (1.83, 2.17), *P* < .001**	1.75 (1.40, 2.20), *P* < .001**
Depression Only^d^ (*n* = 43 452)	1.90 (1.79, 2.01), *P* < .001**	2.15 (2.02, 2.27), *P* < .001**	2.01 (1.84, 2.19), *P* < .001**	1.75 (1.39, 2.19), *P* < .001**^e^
Depression Only 2^f^ (*n* = 5489)	0.51 (0.38, 0.67), *P* < .001**	0.66 (0.50, 0.88), *P* = .005*	0.61 (0.41, 0.91), *P* = .016*	NA

Abbreviations: ACEs, adverse childhood experiences; CI, confidence Interval; HR, hazard ratio; *N*, sample size; NA, not applicable; *P, p*-value; PTSD, post-traumatic stress disorder.

a Main Model: adjusted for demographics (i.e., age, sex, ethnicity, Townsend deprivation index, highest level of education attained).

b Sensitivity analysis 1: adjusted for demographics + lifestyle factors/medical comorbidities (i.e., sleep duration, smoking status, risk group of harm from alcohol consumption, physical activity per week, cardiovascular diseases, traumatic brain injury, hypertension).

c Sensitivity analysis 2: adjusted for demographics + lifestyle factors/medical comorbidities + depression (or ACEs, PTSD, and dissociative disorders when depression is the exposure).

d “Depression only” group refers to participants with depression but without PTSD or dissociative disorders (Depression+/ PTSD–/Dissociative disorders–).

e adjusted for demographics + lifestyle factors/medical comorbidities + ACEs.

f “Depression only 2” group refers to participants without PTSD, dissociative disorders, or depression, who self-reported as part of the online mental health survey that they had no ACEs (Depression+/ACEs–/PTSD–/Dissociative disorders–).

**P < .001, *P < .05.

Self-reported PTSD symptoms were significantly associated with PTSD diagnoses (odds ratio [OR] = 1.30, 95% CI 1.27, 1.32). See the Supplementary Material (Supplementary Figure 1) for boxplots illustrating the distribution of PTSD symptom severity in individuals with and without a PTSD diagnosis. Each one-point increase in the total PTSD severity score was associated with a 9% increase in the risk of dementia (HR_1point_: 1.09; 95% CI 1.06–1.11; *P* < .001). Compared with people without depression, those with depression but without diagnosed PTSD or dissociative disorder had a 2.15-fold increased risk of developing dementia (HR: 2.15; 95% CI 2.02–2.27; *P* < .001), whereas those with depression but without any ACEs, diagnosed PTSD, or dissociative disorder showed a decreased dementia risk (HR: 0.66; 95% CI 0.50–0.88; *P* = .005).

Lastly, our sensitivity analyses revealed that although the associations with dementia were generally attenuated, they remained significant for ACEs, self-reported PTSD symptoms, and depression (Table 2). When adjusting for depression in the models in which ACEs, PTSD, or dissociative disorders were the exposure variables, and when adjusting for ACEs, PTSD, and dissociative disorders in the model in which depression was the exposure variable, self-reported PTSD symptoms and depression remained significantly associated with incident all-cause dementia. Additionally, we conducted a post-hoc power analysis to detect the smallest observed effect size in the main model (i.e., HR = 1.09 for self-reported PTSD symptoms), which yielded an estimated power of 97%.

### Mediation analyses

Our mediation analyses (Table 3) found little evidence to support PTSD diagnosis as a mediator between ACEs and dementia (*P* = .07), whereas self-reported PTSD symptoms significantly mediated the association (*P* < .001), accounting for 75.26% of the excess dementia risk associated with ACEs. Dissociative disorders were not a significant mediator between ACEs and dementia (*P* = .72).

**Table 3.** Mediation analyses

	Total effect		Total natural indirect effect		Overall proportion mediated
	HR (95% CI)	*P*	HR (95% CI)	*P*	
Association between number of types of ACEs (exposure) and dementia (outcome)					
PTSD as a mediator	1.13 (1.03, 1.21)	.01*	1.00 (0.99, 1.00)	.07	NA
Self-reported PTSD symptoms as a mediator^a^	1.11 (1.01, 1.18)	.03*	1.08 (1.06, 1.10)	<.001**	75.26
Dissociative disorders as a mediator	1.12 (1.03, 1.21)	.01*	1.00 (0.999, 1.002)	.72	NA
Depression as a mediator in the association between					
ACEs (exposure) and dementia (outcome)	1.12 (1.03, 1.21)	.01*	1.005 (1.001, 1.01)	.02*	4.51
PTSD (exposure) and dementia (outcome)	2.04 (1.19, 2.90)	< .001**	1.04 (1.02, 1.07)	< .001**	8.42
Self-reported PTSD symptoms (exposure) and dementia (outcome)	1.10 (1.07, 1.12)	< .001**	1.00 (0.999, 1.002)	.15	NA
Dissociative disorders (exposure) and dementia (outcome)	3.93 (2.47, 6.42)	< .001**	1.08 (1.05, 1.13)	< .001**	10.29

Abbreviations: ACEs, adverse childhood experiences; CI, confidence interval; HR, hazard ratio; NA, not applicable; *P*, *p*-value; PTSD = post-traumatic stress disorder.

*Note*: Mediation analyses including self-reported ACEs or self-reported PTSD symptoms included 137 240 participants in the analyses. Adjusted for demographics (i.e., age, sex, ethnicity, Townsend deprivation index, highest level of education attained).

a ACEs and PTSD symptoms were self-reported concurrently during the online mental health survey.

**P < .001, *P < .05.

**Figure 2.**
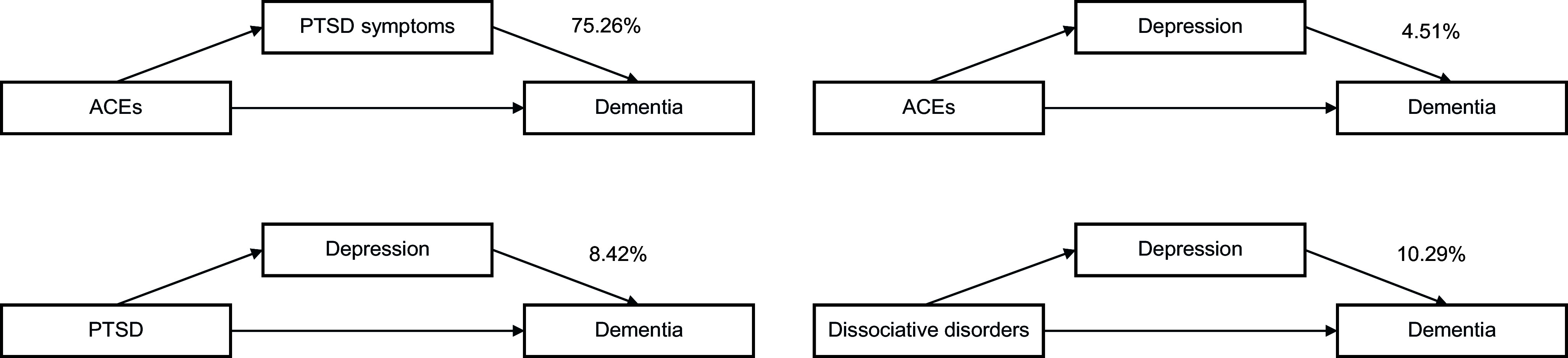
Significant mediators. ACEs, adverse childhood experiences; PTSD, post-traumatic stress disorder. Proposed associations based on mediation analyses. Direct associations were omitted for clarity.

Depression was a significant mediator between ACEs and dementia (*P* = .02; 4.51%) as well as between diagnosed PTSD (*P* < .001; 8.42%) or dissociative disorders (*P* < .001; 10.29%) and dementia, but not between self-reported PTSD symptoms (*P* = .15) and dementia (Figure 2).

## Discussion

In this large UK Biobank cohort, ACEs, PTSD, dissociative disorders, and depression were significantly associated with an increased risk of all-cause dementia. After adjustment for sociodemographic characteristics, we found a dose–response relationship between the number of ACE types and dementia, and between PTSD symptom severity and dementia. The risk of dementia was 2.09, 3.96, and 2.17 times higher for those diagnosed with PTSD, any dissociative disorder, and depression compared to those without these diagnoses. For individuals with depression but without ACEs, PTSD, or dissociative disorders, the associated risk of dementia was reduced by 34%. Self-reported PTSD symptoms accounted for most of the excess dementia risk associated with ACEs. Depression mediated associations between ACEs, diagnosed PTSD, or dissociative disorders and dementia. Thus, self-reported PTSD symptoms were an important mediator of the relationship between ACEs and dementia risk, while depression played a smaller role in the observed associations of ACEs and trauma-related conditions with dementia risk.

Our finding that ACEs are associated with dementia is in line with a recent meta-analysis showing that childhood trauma increases dementia risk by 76% [6]. Another recent study using data from the UK Biobank found that the risk of all-cause dementia in later life was higher in people who experienced childhood trauma compared to adulthood trauma [32], although it included only ACEs related to abuse, not neglect. Moreover, our findings confirm those of a meta-analysis linking PTSD to increased dementia risk [10]. Studies conducted since then have found further evidence that PTSD is a risk factor for dementia [14, 33, 34], with one exception [35]. To the best of our knowledge, no studies to date have looked at PTSD symptom severity, rather than diagnosis, and dementia risk. Including self-reported PTSD symptoms post-hoc was relevant, given the relatively low prevalence of diagnosed PTSD, and importantly, it allowed us to take a dimensional approach (severity score) to complement the categorical one (diagnosis vs. no diagnosis). It is possible that some individuals experience PTSD symptoms at a subclinical level or do not seek help for various reasons and thus are never formally diagnosed. These individuals would be excluded from analyses that rely solely on diagnostic data. In our sample, higher levels of self-reported PTSD symptoms were significantly associated with recorded PTSD diagnoses, though the two were not identical. This suggests that self-report captures meaningful complementary information not reflected in diagnostic records (e.g., subthreshold symptoms, barriers to treatment). By including both measures – ensuring that each was assessed or diagnosed prior to any dementia diagnosis – we aimed to adopt both a dimensional and categorical perspective.

To the best of our knowledge, our study is the first to investigate the relationship between dissociative disorders and dementia. Our findings build on prior studies showing that higher levels of dissociative symptoms are correlated with reduced performance across various cognitive domains [36, 37]. While this is an intriguing result that highlights dissociative disorders as a potential – and potentially modifiable – risk factor for dementia, it must be interpreted with caution. The low prevalence of dissociative disorders in our sample underscores the need for replication in future studies, ideally with higher base rates and more systematic assessments of dissociation-related disorders.

Consistent with prior research [38–41], we found depression to be a significant risk factor for dementia. While earlier studies indicate that later-life depression is associated with, and in fact might be a prodrome of, dementia [39, 42], the recent update from the Lancet Commission on dementia prevention, intervention and care found that depression increases the risk of dementia at all stages of adulthood and therefore classified mid-life depression as a risk factor for dementia [2], which our results further support. Risk of dementia associated with depression, however, was reduced in individuals who reported not having had any ACEs. While this seems counterintuitive, particularly in light of strong evidence from the Lancet Commission on dementia prevention, intervention, and care [2, 39, 42], two possible reasons may account for this observation. First, ACEs might represent a key factor linking both depression and dementia, serving as a common cause that increases vulnerability to both conditions. This would not imply that depression is protective, but rather that the observed association between depression and dementia may attenuate or even reverse once shared aetiology with early-life adversity is accounted for. This perspective aligns with life course models that emphasize how dementia risk is influenced by factors such as lower educational attainment, physical inactivity, substance use (e.g., nicotine and alcohol), and hypertension [2, 39, 42]. Pathways, which themselves may be shaped by ACEs. Second, this finding may reflect a statistical suppressor effect, whereby adjusting for ACEs alters the observed relationship between depression and dementia by removing shared variance. This could result in a negative association in certain subgroups. Again, this would not imply that depression is protective; rather, it underscores the complex interplay between early-life adversity, mental health, and dementia risk. These findings should be interpreted with caution, and future research is needed to replicate our results and further elucidate these relationships.

To the best of our knowledge, our study is the first to systematically explore the interrelationships between ACEs, trauma-related conditions, and depression in their associations with dementia through mediational analyses. Previous studies have used different exposures or have focused on cognitive impairment as the outcome [43, 44]. Their and our findings suggest that ACEs, PTSD, dissociative disorders, and depression likely have both shared and distinct associations leading to cognitive impairment and dementia.

Several mechanisms may explain our results. Early and chronic stress from ACEs, PTSD, dissociative disorders, and depression may cause structural and functional brain changes, increasing vulnerability to neuropathology [45], including dementia. This might occur through prolonged activation of stress- and threat-related pathways [45] and impaired development of brain areas like the hippocampus, amygdala, and frontal cortex [46, 47]. ACEs, PTSD, dissociative disorders, and depression may also hinder cognitive reserve formation [48–50] by reducing engagement in cognitively stimulating activities due to withdrawal from daily life, thereby diminishing the protective buffer against neurodegenerative pathology [51]. This may begin soon after ACEs through impoverished social networks [52] and lower levels of educational attainment [53]. Engagement in repetitive negative thinking (RNT), a transdiagnostic process [54], may contribute to cognitive debt, heightening susceptibility to brain pathology [55]. Higher RNT levels in cognitively intact older adults have been linked to faster declines in global cognition and memory, as well as higher levels of neuropathological markers of Alzheimer’s disease [56]. Our mediation findings suggest that part of the association between ACEs and dementia may operate through self-reported PTSD symptoms and/or depression, and that part of the association between diagnosed PTSD or dissociative disorders and dementia may operate through depression. Certain lifestyle and psychosocial factors across the life course, such as lower educational attainment, social isolation, physical inactivity, smoking, hypertension, and excessive alcohol consumption, have been established as modifiable risk factors for dementia by the Lancet Commission on dementia prevention, intervention, and care [2, 39, 42]. These factors may function as mediators or confounders in the associations between early-life adversity, psychiatric conditions, and dementia risk. While our models adjusted for several of these variables, including education, area-based deprivation, physical activity, alcohol consumption, and smoking, future studies should investigate their potential role as mediators in the pathways linking trauma-related exposures and depression to cognitive decline and dementia.

### Implications

It is important to consider ACEs, PTSD, dissociative disorders, and depression when assessing dementia risk. Evidence is sparse on population-level primary prevention strategies for addressing depression as a risk factor for dementia [57]; an even greater gap exists for ACEs, PTSD, and dissociative disorders. There is currently no evidence on whether clinical interventions, such as trauma-focused therapy, reduce dementia risk. Future research should focus on developing and testing interventions to mitigate dementia risk among individuals with ACEs, trauma-related conditions, and depression. It should also disentangle specific dissociative disorder diagnoses and investigate whether the observed increased risk of dementia is causal or driven by a third variable, such as genetic disposition. Our findings cautiously suggest that ACEs, PTSD, dissociative disorders, and depression may each independently contribute to a higher risk of dementia.

### Strengths and limitations

Strengths of our study include the use of a large, population-based cohort with clinical diagnoses of PTSD, dissociative disorders, and depression, enabling us to adjust for important confounders. Our mediation analyses accounted for the temporal sequence of diagnoses, ensuring that the predictor, mediator, and outcome occurred consecutively. Several limitations should be considered when interpreting our findings. Relatively few cases of PTSD and dissociative disorders were identified, possibly due to underdiagnosis or underreporting in the linked data, thus reducing statistical power. Our sensitivity analyses showed non-significant associations between diagnosed PTSD or dissociative disorders and the risk of dementia. The added exposures likely further reduced the power of these analyses, especially with high collinearity between trauma-related disorders and impaired sleep duration, smoking, cardiovascular diseases, and depression. In contrast, self-reported PTSD symptoms remained significantly associated with dementia in both sensitivity analyses, supporting this association’s robustness. While the overall sample size provided high power for most analyses, predictors with low prevalence, particularly dissociative disorders, may have been underpowered to detect modest effect sizes. Thus, findings related to these conditions should be interpreted with caution and require replication. Moreover, due to power issues, we were not able to adjust the mediation analyses for the remaining exposures. This is particularly relevant given ongoing theoretical debates about the conceptual and empirical overlap between PTSD and dissociation [27, 58–61]. While some researchers view them as closely related but distinct conditions [62, 63], others propose that PTSD may fall within the broader category of dissociative disorders [60]. Future research should pay closer attention to the comorbidity between PTSD and dissociative symptoms and disorders, and investigate newer diagnoses such as the dissociative subtype of PTSD (PTSD-DS), introduced in DSM-5 [58], and complex PTSD (cPTSD), introduced in the recently published ICD-11 [64]. cPTSD includes core PTSD symptoms along with affective dysregulation, a negative self-concept, and difficulties in interpersonal relationships. It has been associated with higher levels of dissociative symptoms than PTSD alone or the absence of PTSD [59]. Additionally, future studies should explore the dimensional nature of trauma-related psychopathology, which may yield more fine-grained insights into how trauma confers risk of dementia. Although our dataset, based on ICD-10, did not include PTSD-DS or cPTSD diagnoses, most individuals with dissociative disorder diagnoses in our sample did not have comorbid PTSD, and vice versa. This supports the rationale for examining these disorders separately. Future research should investigate the role of cPTSD in the risk of dementia, particularly given its relevance to early trauma and dissociation. Additionally, because ACEs were self-reported, recall bias may have influenced our results. People of ethnic minorities and people living with lower socioeconomic circumstances are underrepresented in the UK Biobank, limiting the generalizability of our findings.

## Conclusions

Our study identifies dissociative disorders as a potentially modifiable risk factor for all-cause dementia and provides further evidence that ACEs, PTSD, and depression are risk factors as well. These conditions appear to have both overlapping and distinct associations with increased dementia risk and thus cannot be fully explained by the other investigated exposures. However, due to the low prevalence of some predictors, especially dissociative disorders, our analyses may have been underpowered to detect modest effects. As such, these findings should be interpreted with caution and replicated in future research. Further studies should also attempt to disentangle the underlying mechanisms, both transdiagnostic and disorder-specific, to aid in the development of timely interventions that mitigate the potentially increased risk of dementia associated with ACEs, PTSD, dissociative disorders, and depression.

## Supporting information

10.1192/j.eurpsy.2025.10128.sm001 Günak et al. supplementary material Günak et al. supplementary material

## Data Availability

The data that support the findings of this study are available from the UK Biobank. Access to UK Biobank data can be requested through a standard data access procedure (https://www.ukbiobank.ac.uk/enable-your-research/apply-for-access).

## Supplementary material

The supplementary material for this article can be found at http://doi.org/10.1192/j.eurpsy.2025.10128.

## Analytic code availability

The analytic code is available online (https://osf.io/b28y3/).

Portions of this manuscript were presented at the International Society for Traumatic Stress Studies Annual Meeting, Los Angeles, California, United States, November 3, 2023, and at the Deutscher Psychotherapie Kongress, Berlin, Germany, June 14, 2024.

## References

[1] Prince M, Wimo A, Guerchet M, Ali G-C, Wu Y-T, Prina M. The global impact of dementia: an analysis of prevalence, incidence, cost and trends. London: Alzheimer’s Disease International; 2015.

[2] Livingston G, Huntley J, Liu KY, Costafreda SG, Selbæk G, Alladi S, et al. Dementia prevention, intervention, and care: 2024 report of the Lancet standing Commission. The Lancet 2024:S0140673624012960. 10.1016/S0140-6736(24)01296-0. 39096926

[3] Anda RF, Butchart A, Felitti VJ, Brown DW. Building a framework for global surveillance of the public health implications of adverse childhood experiences. Am J Prev Med 2010;39:93–8. 10.1016/j.amepre.2010.03.015. 20547282

[4] Gilbert R, Widom CS, Browne K, Fergusson D, Webb E, Janson S. Burden and consequences of child maltreatment in high-income countries. The Lancet 2009;373:68–81. 10.1016/S0140-6736(08)61706-7. 19056114

[5] Abouelmagd ME, AbdelMeseh M, Elrosasy A, Eldeeb HA, Nabil Y. Adverse childhood experiences and risk of late-life dementia: a systematic review and meta-analysis. Soc Psychiatry Psychiatr Epidemiol 2024. 10.1007/s00127-024-02676-4. PMC12119739 38717478

[6] Severs E, James T, Letrondo P, Løvland L, Marchant NL, Mukadam N. Traumatic life events and risk for dementia: a systematic review and meta-analysis. BMC Geriatr 2023;23:587. 10.1186/s12877-023-04287-1. 37740188 PMC10517510

[7] Gardner MJ, Thomas HJ, Erskine HE. The association between five forms of child maltreatment and depressive and anxiety disorders: a systematic review and meta-analysis. Child Abuse Negl 2019;96:104082. 10.1016/j.chiabu.2019.104082. 31374447

[8] Messman-Moore TL, Bhuptani PH. A review of the long-term impact of child maltreatment on posttraumatic stress disorder and its comorbidities: an emotion dysregulation perspective. Clin Psychol Sci Pract 2017;24:154–69. 10.1111/cpsp.12193.

[9] Şar V. The many faces of dissociation: opportunities for innovative research in psychiatry. Clin Psychopharmacol Neurosci 2014;12:171–9. 10.9758/cpn.2014.12.3.171. 25598819 PMC4293161

[10] Günak MM, Billings J, Carratu E, Marchant NL, Favarato G, Orgeta V. Post-traumatic stress disorder as a risk factor for dementia: systematic review and meta-analysis. Br J Psychiatry 2020;217:600–8. 10.1192/bjp.2020.150. 32933591

[11] Şar V. Epidemiology of dissociative disorders: an overview. Epidemiol Res Int 2011;2011:1–8. 10.1155/2011/404538.

[12] Bhattarai J “Jackie,” Oehlert ME, Multon KD, Sumerall SW. Dementia and cognitive impairment among U.S. veterans with a history of MDD or PTSD: a retrospective cohort study based on sex and race. J Aging Health 2019;31:1398–422. 10.1177/0898264318781131. 29900802

[13] Flatt JD, Gilsanz P, Quesenberry CP, Albers KB, Whitmer RA. Post-traumatic stress disorder and risk of dementia among members of a health care delivery system. Alzheimers Dement 2018;14:28–34. 10.1016/j.jalz.2017.04.014. 28627380 PMC5729063

[14] Kim H, Park YS, Kim SH, Hurh K, Kim J, Park E-C, et al. Association between stress-related disorders and the risk of dementia using the Korean National Sample Cohort: a matched cohort study. Sci Rep 2023;13:16487. 10.1038/s41598-023-43884-3. 37779110 PMC10543596

[15] Mawanda F, Wallace RB, McCoy K, Abrams TE. PTSD, Psychotropic medication use, and the risk of dementia among US veterans: a retrospective cohort study. J Am Geriatr Soc 2017;65:1043–50. 10.1111/jgs.14756. 28176297

[16] Meziab O, Kirby KA, Williams B, Yaffe K, Byers AL, Barnes DE. Prisoner of war status, posttraumatic stress disorder, and dementia in older veterans. Alzheimers Dement 2014;10:S236–41. 10.1016/j.jalz.2014.04.004. 24924674

[17] Wang T-Y, Wei H-T, Liou Y-J, Su T-P, Bai Y-M, Tsai S-J, et al. Risk for developing dementia among patients with posttraumatic stress disorder: a nationwide longitudinal study. J Affect Disord 2016;205:306–10. 10.1016/j.jad.2016.08.013. 27552595

[18] Yaffe K, Vittinghoff E, Lindquist K, Barnes D, Covinsky KE, Neylan T, et al. Posttraumatic stress disorder and risk of dementia among US veterans. Arch Gen Psychiatry 2010;67:608–608. 10.1001/archgenpsychiatry.2010.61. 20530010 PMC2933793

[19] Yaffe K, Lwi SJ, Hoang TD, Xia F, Barnes DE, Maguen S, et al. Military-related risk factors in female veterans and risk of dementia. Neurology 2019;92. 10.1212/WNL.0000000000006778. 30541865 PMC6340384

[20] Flory JD, Yehuda R. Comorbidity between post-traumatic stress disorder and major depressive disorder: alternative explanations and treatment considerations. Dialogues Clin Neurosci 2015;17:141–50. 10.31887/DCNS.2015.17.2/jflory. 26246789 PMC4518698

[21] Davis KAS, Coleman JRI, Adams M, Allen N, Breen G, Cullen B, et al. Mental health in UK Biobank – development, implementation and results from an online questionnaire completed by 157 366 participants: a reanalysis. BJPsych Open 2020;6:e18. 10.1192/bjo.2019.100. 32026800 PMC7176892

[22] Townsend P, Phillimore P, Beattie A. Health and deprivation: inequality and the North. 1st ed. London: Routledge; 2023. 10.4324/9781003368885.

[23] Craig CL, Marshall AL, Sjöström M, Bauman AE, Booth ML, Ainsworth BE, et al. International physical activity questionnaire: 12-country reliability and validity: Med Sci Sports Exerc 2003;35:1381–95. 10.1249/01.MSS.0000078924.61453.FB. 12900694

[24] NICE. Overview | Alcohol-use disorders: prevention | Guidance | NICE 2010. https://www.nice.org.uk/guidance/ph24 (accessed May 24, 2024).

[25] Glaesmer H, Schulz A, Häuser W, Freyberger H, Brähler E, Grabe H-J. Der Childhood Trauma Screener (CTS) – Entwicklung und Validierung von Schwellenwerten zur Klassifikation. Psychiatr Prax 2013;40:220–6. 10.1055/s-0033-1343116. 23564353

[26] Bernstein D, L Fink, Handelsman L, Foote J, Lovejoy M, Wenzel K, et al. Initial reliability and validity of a new retrospective measure of child abuse and neglect. Am J Psychiatry 1994;151:1132–6. 10.1176/ajp.151.8.1132. 8037246

[27] World Health Organization. The ICD-10 Classification of Mental and Behavioural Disorders. 1993.

[28] Wilkins KC, Lang AJ, Norman SB. Synthesis of the psychometric properties of the PTSD checklist (PCL) military, civilian, and specific versions. Depress Anxiety 2011;28:596–606. 10.1002/da.20837. 21681864 PMC3128669

[29] Sareen J. Posttraumatic stress disorder in adults: impact, comorbidity, risk factors, and treatment. Can J Psychiatry 2014;59:460–7. 10.1177/070674371405900902. 25565692 PMC4168808

[30] Stansfeld S, Rasul F. Psychosocial factors, depression and illness. In: Steptoe A, editor. Depression and physical illness. 1st ed. Cambridge, UK: Cambridge University Press; 2006, pp. 19–50. 10.1017/CBO9780511544293.003.

[31] Shi B, Choirat C, Coull BA, VanderWeele TJ, Valeri L. CMAverse: A suite of functions for reproducible causal mediation analyses. Epidemiology 2021;32:e20–2. 10.1097/EDE.0000000000001378. 34028370

[32] Xie Z, Li M, Sun H, Zhou C, Fu C, Wang Q, et al. Childhood, adulthood, and cumulative traumatic events experienced from childhood to adulthood and dementia risk: a population-based cohort study. J Public Health 2023;33(8), 1625–1635. 10.1007/s10389-023-02140-8.

[33] Bergman BP, Mackay DF, Pell JP. Dementia in Scottish military veterans: early evidence from a retrospective cohort study. Psychol Med 2021:1–6. 10.1017/S0033291721002440. PMC9975994 34165055

[34] Song H, Sieurin J, Wirdefeldt K, Pedersen NL, Almqvist C, Larsson H, et al. Association of stress-related disorders with subsequent neurodegenerative diseases. JAMA Neurol 2020;77:700–9. 10.1001/JAMANEUROL.2020.0117. 32150226 PMC7063561

[35] Islamoska S, Hansen ÅM, Ishtiak-Ahmed K, Garde AH, Andersen PK, Garde E, et al. Stress diagnoses in midlife and risk of dementia: a register-based follow-up study. Aging & Mental Health 2020;25(6), 1151–1160. 10.1080/13607863.2020.1742656. 32233797

[36] McKinnon MC, Boyd JE, Frewen PA, Lanius UF, Jetly R, Richardson JD, et al. A review of the relation between dissociation, memory, executive functioning and social cognition in military members and civilians with neuropsychiatric conditions. Neuropsychologia 2016;90:210–34. 10.1016/j.neuropsychologia.2016.07.017. 27444881

[37] Alexis V, Géraldine T, David C, Camille R, Wissam E-H. Association between cognitive impairments and dissociation: A PRISMA systematic review. Eur J Trauma Dissociation 2023;7:100341. 10.1016/j.ejtd.2023.100341.

[38] Kuring JK, Mathias JL, Ward L. Risk of Dementia in persons who have previously experienced clinically-significant depression, anxiety, or PTSD: a systematic review and meta-analysis. J Affect Disord 2020;274:247–61. 10.1016/j.jad.2020.05.020. 32469813

[39] Livingston G, Huntley J, Sommerlad A, Ames D, Ballard C, Banerjee S, et al. Dementia prevention, intervention, and care: 2020 report of the Lancet Commission. The Lancet 2020;396:413–46. 10.1016/S0140-6736(20)30367-6. PMC7392084 32738937

[40] Ownby RL, Crocco E, Acevedo A, John V, Loewenstein D. Depression and risk for Alzheimer disease: systematic review, meta-analysis, and metaregression analysis. Arch Gen Psychiatry 2006;63:530. 10.1001/archpsyc.63.5.530. 16651510 PMC3530614

[41] Stafford J, Chung WT, Sommerlad A, Kirkbride JB, Howard R. Psychiatric disorders and risk of subsequent dementia: systematic review and meta-analysis of longitudinal studies. Int J Geriatr Psychiatry 2022;37:gps.5711. 10.1002/gps.5711. PMC9325434 35460299

[42] Livingston G, Sommerlad A, Orgeta V, Costafreda SG, Huntley J, Ames D, et al. Dementia prevention, intervention, and care. The Lancet 2017;390:2673–734. 10.1016/S0140-6736(17)31363-6. 28735855

[43] Singh A, Zeig-Owens R, Rabin L, Schwartz T, Webber MP, Appel D, et al. PTSD and depressive symptoms as potential mediators of the association between World Trade Center Exposure and Subjective Cognitive Concerns in Rescue/Recovery Workers. Int J Environ Res Public Health 2020;17:5683. 10.3390/ijerph17165683. 32781591 PMC7460046

[44] Cohn-Schwartz E, Hoffman Y, Shrira A. Reciprocal associations of posttraumatic stress symptoms and cognitive decline in community-dwelling older adults: the mediating role of depression. Int Psychogeriatr 2024;36:119–29. 10.1017/S1041610222000357. 35543414

[45] McEwen BS. Physiology and neurobiology of stress and adaptation: central role of the brain. Physiol Rev 2007;87:873–904. 10.1152/physrev.00041.2006. 17615391

[46] Lupien SJ, McEwen BS, Gunnar MR, Heim C. Effects of stress throughout the lifespan on the brain, behaviour and cognition. Nat Rev Neurosci 2009;10:434–45. 10.1038/nrn2639. 19401723

[47] Nilaweera D, Freak-Poli R, Ryan J. The impact of psychological stress and trauma on later-life cognitive function and dementia. J Gerontol Geriatr 2019;67:114–22.

[48] Stern Y. Influence of education and occupation on the incidence of Alzheimer’s disease. JAMA J Am Med Assoc 1994;271:1004–1004. 10.1001/jama.1994.03510370056032. 8139057

[49] Stern Y. What is cognitive reserve? Theory and research application of the reserve concept. J Int Neuropsychol Soc 2002;8:448–60. 10.1017/S1355617702813248. 11939702

[50] Tucker AM, Stern Y. Cognitive reserve in aging. Curr Alzheimer Res 2011;8:354–60. 10.2174/156720511795745320. 21222591 PMC3135666

[51] Almeida-Meza P, Steptoe A, Cadar D. Markers of cognitive reserve and dementia incidence in the English Longitudinal Study of Ageing. Br J Psychiatry 2021;218:243–51. 10.1192/bjp.2020.54. 32223764 PMC7529639

[52] McCrory E, Foulkes L, Viding E. Social thinning and stress generation after childhood maltreatment: a neurocognitive social transactional model of psychiatric vulnerability. Lancet Psychiatry 2022;9:828–37. 10.1016/S2215-0366(22)00202-4. 35926524

[53] Blodgett C, Lanigan JD. The association between adverse childhood experience (ACE) and school success in elementary school children. Sch Psychol Q 2018;33:137–46. 10.1037/spq0000256. 29629790

[54] Ehring T, Watkins ER. Repetitive negative thinking as a transdiagnostic process. Int J Cogn Ther 2008;1:192–205. 10.1521/ijct.2008.1.3.192.

[55] Marchant NL, Howard RJ. Cognitive debt and Alzheimer’s disease. J Alzheimers Dis 2015;44:755–70. 10.3233/JAD-141515. 25362035

[56] Marchant NL, Lovland LR, Jones R, Pichet Binette A, Gonneaud J, Arenaza-Urquijo EM, et al. Repetitive negative thinking is associated with amyloid, tau, and cognitive decline. Alzheimers Dement 2020;16:1054–64. 10.1002/alz.12116. 32508019

[57] Walsh S, Wallace L, Kuhn I, Mytton O, Lafortune L, Wills W, et al. Population-level interventions for the primary prevention of dementia: a complex evidence review. eClinicalMedicine 2024;70:102538. 10.1016/j.eclinm.2024.102538. 38495526 PMC10940136

[58] American Psychiatric Association. Diagnostic and statistical manual of mental disorders, Washington, DC: American Psychiatric Association. 5th ed; 2013. 10.1176/appi.books.9780890425596.744053.

[59] Hyland P, Shevlin M, Fyvie C, Cloitre M, Karatzias T. The relationship between ICD-11 PTSD, complex PTSD and dissociative experiences. J Trauma Dissociation 2020;21:62–72. 10.1080/15299732.2019.1675113. 31583967

[60] Nijenhuis ERS. Ten reasons for conceiving and classifying posttraumatic stress disorder as a dissociative disorder. Eur J Trauma Dissociation 2017;1:47–61. 10.1016/j.ejtd.2017.01.001.

[61] Schalinski I, Teicher MH, Nischk D, Hinderer E, Müller O, Rockstroh B. Type and timing of adverse childhood experiences differentially affect severity of PTSD, dissociative and depressive symptoms in adult inpatients. BMC Psychiatry 2016;16:295. 10.1186/s12888-016-1004-5. 27543114 PMC4992284

[62] Dalenberg C, Carlson EB. Dissociation in posttraumatic stress disorder part II: How theoretical models fit the empirical evidence and recommendations for modifying the diagnostic criteria for PTSD. Psychol Trauma Theory Res Pract Policy 2012;4:551–9. 10.1037/a0027900.

[63] Kihlstrom JF. Dissociative disorders. Annu Rev Clin Psychol 2005;1:227–53. 10.1146/annurev.clinpsy.1.102803.143925. 17716088

[64] Maercker A, Brewin CR, Bryant RA, Cloitre M, Van Ommeren M, Jones LM, et al. Diagnosis and classification of disorders specifically associated with stress: proposals for ICD-11. World Psychiatry 2013;12:198–206. 10.1002/wps.20057. 24096776 PMC3799241

