# Supplementary material for: Impact of adverse childhood experiences, post-traumatic stress disorder, dissociative disorders, and depression on dementia risk: a prospective analysis of associations and mediation in the UK Biobank cohort
Source: Eur Psychiatry. 2025 Oct 29;68(1):e166. doi: 10.1192/j.eurpsy.2025.10128 (PMC12646123; doi:10.1192/j.eurpsy.2025.10128)
Supplement: Günak et al. supplementary material [file S0924933825101284sup001.docx]

**Online Supplementary Material:**

**Impact of adverse childhood experiences, post-traumatic stress disorder, dissociative disorders, and depression on dementia risk: A prospective analysis of associations and mediation in the UK Biobank cohort**

Mia Maria Günak, MSc,^a,b,c^ Thomas Ehring, PhD,^a,d^ Vasiliki Orgeta, PhD,^c^

Frederick K. Ho, PhD^b^

^a^Department of Psychology, Division of Clinical Psychology and Psychological Treatment, LMU Munich, Munich, Germany

^b^School of Health & Wellbeing, University of Glasgow, Glasgow, United Kingdom

^c^Division of Psychiatry, Faculty of Brain Sciences, University College London, London, United Kingdom

^d^German Center for Mental Health (DZPG), Munich, Germany

Corresponding Author: Mia Maria Günak, MSc, Department of Psychology, Division of Clinical Psychology and Psychological Treatment, LMU Munich, Leopoldstrasse 13, 80802 Munich, Germany.

E-mail: [mia.guenak@psy.lmu.de](mailto:mia.guenak@psy.lmu.de)

**eTable 1.** Adverse Childhood Experiences (ACEs) by Group

**eTable 2.** Dementia Outcome by Group

**eTable 3.** Dementia Outcome by Number of Types of Adverse Childhood Experiences (ACEs) (n = 137 631)

**eFigure 1.** Distribution of Self-Reported PTSD Symptoms by PTSD Diagnosis

**eMethods.** Used R packages and versions

**eTable 1. Adverse Childhood Experiences (ACEs) by Group**

|  | **Overall**  **(n *=* 434 215)** | **Comparison group**  **(n *=* 389 516)^a^** | **ACEs**  **(n *=* 45 536)^b^** | **PTSD**  **(n *=* 941)** | **Dissociative disorders**  **(n *=* 325)** | **Depression**  **(n *=* 44 140)** | **Depression only**  **(n *=* 43 452)^c^** |
| --- | --- | --- | --- | --- | --- | --- | --- |
| **Types of ACEs**  **n (%)** | 45 536 / 137 631 (33.09) | 40 081 / 126 577 (31.67) | 45 536 / 45 536 (100.00) | 111 / 189 (58.73) | 47 / 79 (59.49) | 5381 / 10 914 (49.30) | 5301 / 10 790 (49.13) |
| 0 | 92 095 / 137 631 (66.91) | 86 496 / 126 577 (68.33) | 0 / 45 536 (0.00) | 78 / 189 (41.27) | 32 / 79 (40.51) | 5533 / 10 914 (50.70) | 5489 / 10 790 (50.87) |
| 1 | 27 975 / 137 631 (20.33) | 25 286 / 126 577 (19.98) | 27 975 / 45 536 (61.43) | 35 / 189 (18.52) | 18 / 79 (22.78) | 2660 / 10 914 (24.37) | 2636 / 10 790 (24.43) |
| 2 | 10 422 / 137 631 (7.57) | 9014 / 126 577 (7.12) | 10 422 / 45 536 (22.89) | 24 / 189 (12.70) | 10 / 79 (12.66) | 1385 / 10 914 (12.69) | 1374 / 10 790 (12.73) |
| 3 | 4636 / 137 631 (3.37) | 3860 / 126 577 (3.05) | 4636 / 45 536 (10.18) | 21 / 189 (11.11) | 11 / 79 (13.92) | 766 / 10,914 (7.02) | 747 / 10 790 (6.92) |
| 4 | 1898 / 137 631 (1.38) | 1485 / 126 577 (1.17) | 1898 / 45 536 (4.17) | 17 / 189 (8.99) | 3 / 79 (3.80) | 408 / 10 914 (3.74) | 394 / 10 790 (3.65) |
| 5 | 605 / 137 631 (0.44) | 436 / 126 577 (0.34) | 605 / 45 536 (1.33) | 14 / 189 (7.41) | 5 / 79 (6.33) | 162 / 10 914 (1.48) | 150 / 10 790 (1.39) |
| Physical abuse | 11 426 / 140 478 (8.13) | 9735 / 129 036 (7.54) | 10 914 / 45 536 (23.97) | 54 / 200 (27.00) | 18 / 82 (21.95) | 1660 / 11 297 (14.69) | 1620 / 11 164 (14.51) |
| Emotional abuse | 13 215 / 140 412 (9.41) | 10 955 / 128 980 (8.49) | 12 667 / 45 536 (27.82) | 64 / 200 (32.00) | 21 / 81 (25.93) | 2226 / 11 288 (19.72) | 2178 / 11 155 (19.52) |
| Sexual abuse | 12 161 / 139 202 (8.74) | 10 524 / 127 956 (8.22) | 12 006 / 45 536 (26.37) | 52 / 194 (26.80) | 26 / 81 (32.10) | 1604 / 11 102 (14.45) | 1562 / 10 975 (14.23) |
| Physical neglect | 7876 / 139 856 (5.63) | 6782 / 128 464 (5.28) | 7586 / 45 536 (16.66) | 40 / 201 (19.90) | 13 / 82 (15.85) | 1078 / 11 247 (9.58) | 1044 / 11 113 (9.39) |
| Emotional neglect | 31 222 / 140 259 (22.26) | 27 020 / 128 831 (20.97) | 30 171 / 45 536 (66.26) | 87 / 202 (43.07) | 33 / 83 (39.76) | 4152 / 11 283 (36.80) | 4085 / 11 147 (36.65) |

ACEs = adverse childhood experiences; PTSD = post-traumatic stress disorder; N = sample size.

The categories are not mutually exclusive. Only available at follow-up, in 2017, as part of the online mental health survey.

^a^“Comparison group” refers to participants without PTSD, dissociative disorders, or depression (PTSD–/Dissociative disorders–/Depression–).

^b^“ACEs” group refers to participants who self-reported that they had at least one type of ACEs as part of the online mental health survey (ACEs+).

^c^“Depression only” group refers to participants with depression but without PTSD or dissociative disorders (Depression+/ PTSD–/Dissociative disorders–).

**eTable 2. Dementia Outcome by Group**

|  | **Overall**  **(n = 434 215)** | **Comparison group^a^**  **(n = 389 516)** | **Comparison group 2^b^**  **(n = 86 496)** | **ACEs^c^**  **(n = 45 536)** | **PTSD**  **(n = 941)** | **Dissociative disorders**  **(n = 325)** | **Depression**  **(n = 44 140)** | **Depression only^d^**  **(n = 43 452)** | **Depression only 2^e^ (n = 5489)** |
| --- | --- | --- | --- | --- | --- | --- | --- | --- | --- |
| **Dementia**  **(n, %)** | 8118 (1.87) | 6703 (1.72) | 454 (0.52) | 266 (0.58) | 22 (2.34) | 20 (6.15) | 1397 (3.16) | 1373 (3.16) | 48 (0.87) |

N = sample size; ACEs = adverse childhood experiences; PTSD = post-traumatic stress disorder. The categories are not mutually exclusive.

^a^“Comparison group” refers to participants without PTSD, dissociative disorders, or depression (PTSD–/Dissociative disorders–/Depression–).

^b^“Comparison group 2” refers to participants without PTSD, dissociative disorders, or depression, who self-reported as part of the online mental health survey that they had no ACEs (ACEs–/PTSD–/Dissociative disorders–/Depression–).

^c^“ACEs” group refers to participants who self-reported that they had at least one type of ACEs, as part of the online mental health survey (ACEs+).

^d^“Depression only” group refers to participants with depression but without PTSD or dissociative disorders (Depression+/ PTSD–/Dissociative disorders–).

^e^“Depression only 2” group refers to participants without PTSD, dissociative disorders, or depression, who self-reported as part of the online mental health survey that they had no ACEs (Depression+/ACEs–/PTSD–/Dissociative disorders–).

**eTable 3. Dementia Outcome by Number of Types of Adverse Childhood Experiences (ACEs)** **(n = 137 631)**

| **Number of ACE types** | **0**  **(n = 92 095)** | **1**  **(n = 27 975)** | **2**  **(n = 10 422)** | **3**  **(n = 4636)** | **4**  **(n = 1898)** | **5**  **(n = 605)** |
| --- | --- | --- | --- | --- | --- | --- |
| **Dementia**  **(n, %)** | 503 (0.55) | 158 (0.56) | 64 (0.61) | 30 (0.65) | 12 (0.63) | 2 (0.33) |

ACEs = adverse childhood experiences; N = sample size.

The following types of ACEs are included: emotional neglect, physical neglect, emotional abuse, physical abuse, and sexual abuse.

**eFigure 1. Distribution of Self-Reported PTSD Symptoms by PTSD Diagnosis**


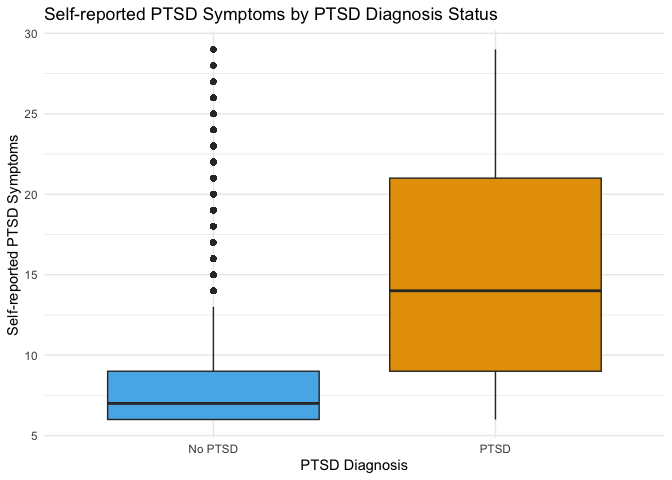


**eMethods. Used R Packages and Versions**

R version 4.2.0 (2022-04-22)

Platform: x86_64-apple-darwin17.0 (64-bit)

Running under: macOS 14.4

Matrix products: default

LAPACK: /Library/Frameworks/R.framework/Versions/4.2/Resources/lib/libRlapack.dylib

locale:

[1] de_DE.UTF-8/de_DE.UTF-8/de_DE.UTF-8/C/de_DE.UTF-8/de_DE.UTF-8

attached base packages:

[1] stats graphics grDevices utils datasets methods base

other attached packages:

[1] dplyr_1.1.2 CMAverse_0.1.0

loaded via a namespace (and not attached):

[1] Rcpp_1.0.8.3 MetaUtility_2.1.2 msm_1.7 mvtnorm_1.1-3

[5] lattice_0.20-45 tidyr_1.3.0 zoo_1.8-10 digest_0.6.29

[9] utf8_1.2.2 R6_2.5.1 backports_1.4.1 survey_4.1-1

[13] evaluate_0.15 ggplot2_3.4.2 pillar_1.9.0 rlang_1.1.1

[17] multcomp_1.4-20 rstudioapi_0.13 car_3.0-13 Matrix_1.4-1

[21] rmarkdown_2.19 mathjaxr_1.6-0 splines_4.2.0 stringr_1.5.0

[25] igraph_1.3.1 munsell_0.5.0 broom_1.0.3 compiler_4.2.0

[29] xfun_0.31 pkgconfig_2.0.3 EValue_4.1.3 mitools_2.4

[33] htmltools_0.5.4 nnet_7.3-17 tidyselect_1.2.0 tibble_3.2.1

[37] expm_0.999-7 codetools_0.2-18 simex_1.8 fansi_1.0.3

[41] withr_2.5.0 MASS_7.3-56 SuppDists_1.1-9.7 grid_4.2.0

[45] DBI_1.1.3 nlme_3.1-157 gtable_0.3.0 lifecycle_1.0.3

[49] magrittr_2.0.3 metafor_3.8-1 scales_1.2.0 metadat_1.2-0

[53] cli_3.6.0 stringi_1.7.6 carData_3.0-5 mice_3.14.0

[57] generics_0.1.2 vctrs_0.6.2 boot_1.3-28 sandwich_3.0-2

[61] TH.data_1.1-1 tools_4.2.0 ggdag_0.2.7 medflex_0.6-7

[65] glue_1.6.2 purrr_1.0.1 abind_1.4-5 fastmap_1.1.0

[69] survival_3.5-0 yaml_2.3.5 colorspace_2.0-3 tidygraph_1.2.2

[73] knitr_1.39

R version 4.2.0 (2022-04-22)

Platform: x86_64-apple-darwin17.0 (64-bit)

Running under: macOS 14.4

Matrix products: default

LAPACK: /Library/Frameworks/R.framework/Versions/4.2/Resources/lib/libRlapack.dylib

locale:

[1] de_DE.UTF-8/de_DE.UTF-8/de_DE.UTF-8/C/de_DE.UTF-8/de_DE.UTF-8

attached base packages:

[1] stats graphics grDevices utils datasets methods base

other attached packages:

[1] ggfortify_0.4.15 ranger_0.14.1 survival_3.5-0 forcats_0.5.1 stringr_1.5.0

[6] purrr_1.0.1 readr_2.1.2 tidyr_1.3.0 tibble_3.2.1 ggplot2_3.4.2

[11] tidyverse_1.3.2 gtsummary_1.7.0 psych_2.2.5 dplyr_1.1.2 CMAverse_0.1.0

loaded via a namespace (and not attached):

[1] TH.data_1.1-1 googledrive_2.0.0 colorspace_2.0-3 ellipsis_0.3.2

[5] fs_1.5.2 rstudioapi_0.13 mice_3.14.0 fansi_1.0.3

[9] mvtnorm_1.1-3 lubridate_1.8.0 mathjaxr_1.6-0 xml2_1.3.3

[13] codetools_0.2-18 splines_4.2.0 simex_1.8 mnormt_2.0.2

[17] medflex_0.6-7 knitr_1.39 SuppDists_1.1-9.7 jsonlite_1.8.0

[21] gt_0.8.0 broom_1.0.3 dbplyr_2.3.0 compiler_4.2.0

[25] httr_1.4.3 backports_1.4.1 assertthat_0.2.1 Matrix_1.4-1

[29] fastmap_1.1.0 gargle_1.2.1 survey_4.1-1 cli_3.6.0

[33] htmltools_0.5.4 tools_4.2.0 igraph_1.3.1 gtable_0.3.0

[37] glue_1.6.2 Rcpp_1.0.8.3 msm_1.7 carData_3.0-5

[41] cellranger_1.1.0 vctrs_0.6.2 ggdag_0.2.7 nlme_3.1-157

[45] broom.helpers_1.11.0 EValue_4.1.3 xfun_0.31 rvest_1.0.3

[49] lifecycle_1.0.3 googlesheets4_1.0.1 MASS_7.3-56 zoo_1.8-10

[53] scales_1.2.0 tidygraph_1.2.2 hms_1.1.1 parallel_4.2.0

[57] sandwich_3.0-2 expm_0.999-7 metafor_3.8-1 yaml_2.3.5

[61] gridExtra_2.3 sass_0.4.4 labelled_2.10.0 stringi_1.7.6

[65] boot_1.3-28 rlang_1.1.1 pkgconfig_2.0.3 commonmark_1.8.1

[69] evaluate_0.15 lattice_0.20-45 tidyselect_1.2.0 magrittr_2.0.3

[73] R6_2.5.1 generics_0.1.2 multcomp_1.4-20 DBI_1.1.3

[77] pillar_1.9.0 haven_2.5.0 withr_2.5.0 abind_1.4-5

[81] nnet_7.3-17 modelr_0.1.10 crayon_1.5.1 car_3.0-13

[85] utf8_1.2.2 tmvnsim_1.0-2 tzdb_0.3.0 rmarkdown_2.19

[89] grid_4.2.0 metadat_1.2-0 readxl_1.4.1 reprex_2.0.2

[93] digest_0.6.29 MetaUtility_2.1.2 munsell_0.5.0 mitools_2.4

The analytic code is available online (<https://osf.io/b28y3/>).
